# Pyogenic Liver Abscess and Delayed Massive Gastrointestinal Bleeding Following Endoscopic Retrograde Cholangiopancreatography (ERCP): Association of Two Rare Complications

**DOI:** 10.7759/cureus.30374

**Published:** 2022-10-17

**Authors:** Francisco Vara-Luiz, Fábio Pé D’Arca Barbosa, Ana Albuquerque, Eduardo Fernandes, Ana Valada Marques, Vanda Spencer, Gonçalo Nunes, Jorge Fonseca

**Affiliations:** 1 Gastroenterology, Hospital Garcia de Orta, Almada, PRT; 2 Grupo de Patologia Médica, Nutrição e Exercício Clínico (PaMNEC), Centro de Investigação Interdisciplinar Egas Moniz (CiiEM), Almada, PRT; 3 Internal Medicine, Hospital Garcia de Orta, Almada, PRT; 4 Radiology, Hospital Garcia de Orta, Almada, PRT

**Keywords:** post-sphincterotomy bleeding, biliary stent, ercp, gastrointestinal bleeding, liver abscess

## Abstract

A liver abscess (LA) is the most common type of visceral abscess. While biliary tract disorders are its most common etiology, clinicians should also consider less frequent causes such as iatrogenic complications due to certain interventions. One of these unusual causes is related to endoscopic retrograde cholangiopancreatography (ERCP) with endoscopic sphincterotomy (ES), a usually safe procedure that carries some risk of complications. We present the case of a 71-year-old female with a history of choledocholithiasis who underwent ERCP with ES without any immediate complications; she was discharged after 24 hours and readmitted three days later to the emergency room with fever and abdominal pain. An abdominal CT showed a liver abscess. Blood cultures were positive for *Escherichia coli*, *Streptococcus anginosus,* and *Enterococcus faecalis*, and the patient was started on directed antibiotic therapy with ampicillin, benzylpenicillin, and metronidazole. On day 17, due to hematochezia with hemodynamic instability, an urgent upper gastrointestinal endoscopy was performed, which revealed late post-ES bleeding, refractory to conventional endoscopic therapy. An ERCP was performed to control the bleeding by using a biliary fully covered self-expandable metal stent (FCSEMS), which was removed four weeks later. The follow-up CT showed a significant reduction of LA and the patient was discharged. This case highlights the association of two uncommon complications of ERCP: a LA and a major late post-ES bleeding. Clinicians should maintain a high index of suspicion for these complications in daily practice.

## Introduction

A liver abscess (LA) is the most common type of visceral abscess, with an estimated mortality rate ranging from 10 to 40% [1]. There are several risk factors associated with pyogenic liver abscess (PLA), namely diabetes mellitus, cirrhosis, immunocompromised state, use of proton pump inhibitors (PPI), male gender, and age. While the most common causes are found in the biliary tract [2], other routes of infection include hematogenic spread, direct extension, and iatrogenic causes. Endoscopic retrograde cholangiopancreatography (ERCP) with endoscopic sphincterotomy (ES) is the gold standard for the management of bile duct stones [3]. However, ES carries some risk of complications, such as upper gastrointestinal bleeding (UGIB), and, recently, it has been associated with LA formation [4]. We report a case of PLA and a late massive UGIB following ERCP with ES.

## Case presentation

**Figure 1:**
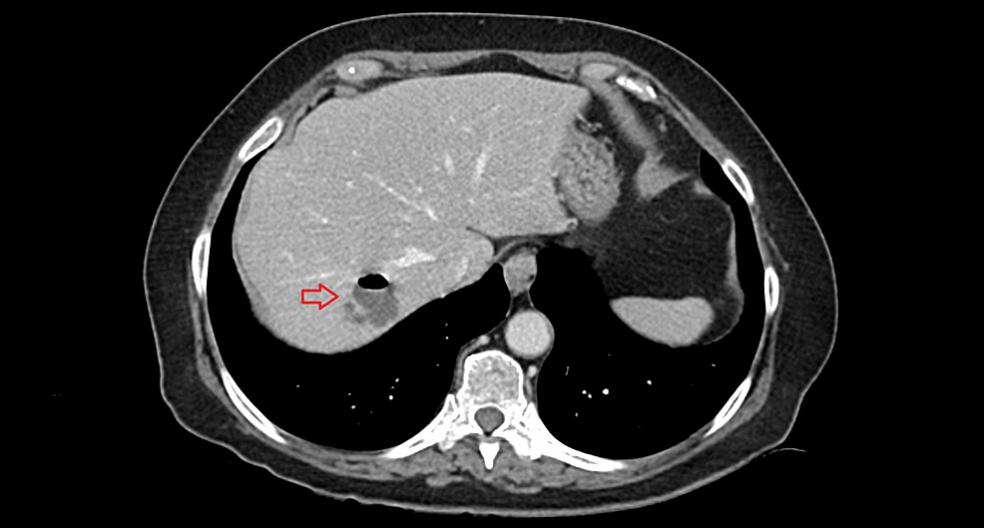
Abdominal CT showing a liver abscess (red arrow) CT: computed tomography

A 71-year-old Caucasian female with a past medical history of dyspeptic symptoms relieved with PPI, and choledocholithiasis, underwent ERCP with ES and biliary stone extraction without any immediate complications and was discharged after 24 hours. Two days after the procedure, the patient started to experience fever and abdominal pain in the right upper quadrant with no radiation and no correlation with meals and presented to the emergency room the following day. On physical examination, she was hemodynamically stable, with a fever (temperature of 38.5 ºC) and tenderness in the right upper quadrant. Laboratory evaluation showed mild leukocytosis with neutrophilia and increased C-reactive protein. An abdominal CT revealed a 33 x 35-mm LA (Figure 1) and the patient was admitted for stabilization; empiric treatment with cefuroxime and metronidazole was initiated.

**Figure 2:**
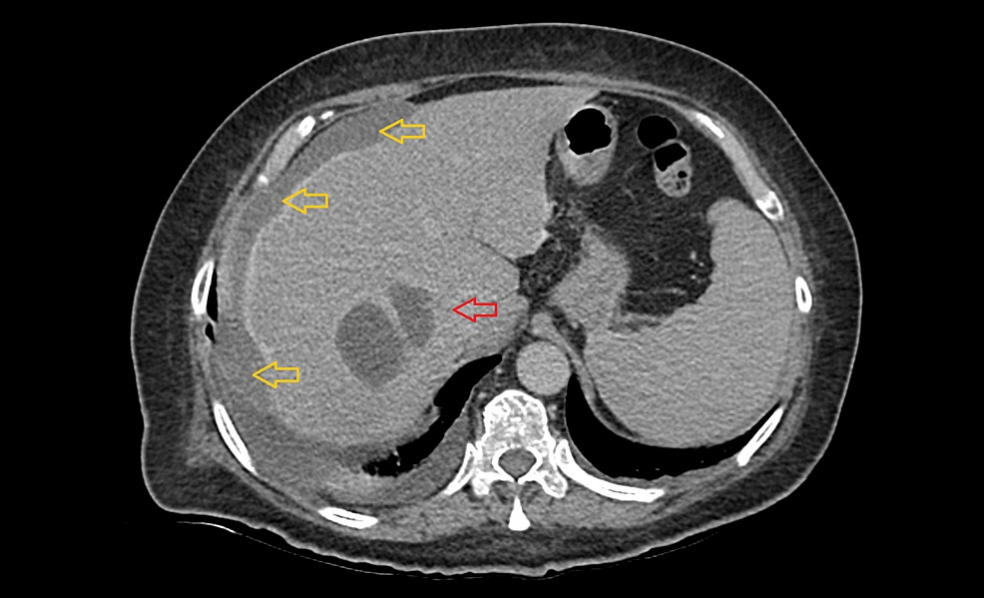
Abdominal CT showing the same liver abscess (red arrow) and an iatrogenic hemoperitoneum (yellow arrows) CT: computed tomography

Blood cultures were positive for *Escherichia coli, Streptococcus anginosus, *and* Enterococcus faecalis*, and antibiotics were changed according to the antibiotic sensitivity test: *Streptococcus anginosus* was susceptible to penicillin, unlike *Enterococcus faecalis*, which was resistant to penicillin but susceptible to ampicillin. For this reason, after discussing the case with the hospital infection committee, the patient was started on ampicillin and benzylpenicillin and metronidazole was maintained. Transesophageal echocardiography excluded infective endocarditis. On day 12 of hospitalization, ultrasound-guided drainage was performed, which resulted in iatrogenic hemoperitoneum, confirmed with an abdominal CT (Figure 2) and requiring red blood cell transfusions after which the patient stabilized. Given the difficulty in access to the LA for drainage, no other attempt was made, and the patient's condition slowly improved during hospitalization.

**Figure 3:**
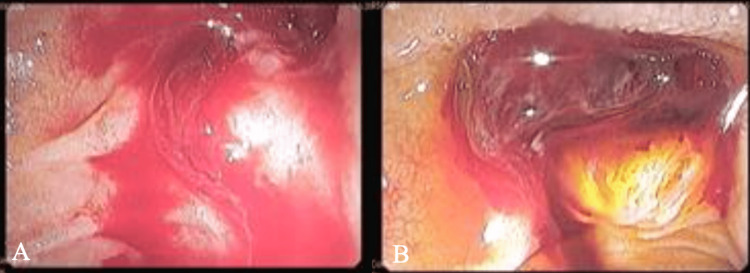
Upper gastrointestinal endoscopy showing active bleeding in the duodenum (A) and a visible juxtapapillary clot (B)

However, on day 17, the patient started to have hematochezia with hemodynamic instability, requiring urgent upper gastrointestinal endoscopy, which revealed massive active bleeding from the site of previous ES (Figure 3), which was refractory to local adrenalin injection and sclerotherapy with polidocanol. A new ERCP was performed to control UGIB with the placement of a biliary fully covered self-expandable metal stent (FCSEMS), which resulted in clinical and analytical improvement. The biliary stent was removed four weeks later with no associated complications or rebleeding. Risk factors that predispose to bleeding, such as excess alcohol use, liver, kidney, and hematologic diseases, and disorders of hemostasis were all excluded.

Five weeks after the beginning of directed therapy, a control abdominal CT showed a significant reduction of the LA (9 x 2 mm). On day 54, the patient was discharged home with oral amoxicillin-clavulanate and metronidazole that was maintained for one more month. A follow-up abdominal CT after the conclusion of bacteriologic therapy showed complete resolution of the LA.

## Discussion

PLA is an uncommon condition characterized by solitary or multiple purulent collections within the liver. Most of them are polymicrobial, with *Escherichia coli*, *Streptococcus spp*. and *Klebsiella pneumoniae *being the common causative organisms [5]. Most cases are caused by ascending infection from biliary tract disorders. However, it is important to consider less frequent causes. Drainage and antibiotic therapy are standard components of treatment [2].

Our patient had multiple risk factors for PLA: advanced age, chronic use of PPI, and recent ERCP with ES, which, probably had a major role in this case. ERCP with ES is considered a safe procedure, with increasing indications [6]. However, there are some complications that need to be addressed. Prior ES promotes duodenal-biliary reflux by breaking the barrier between the hepatobiliary system and duodenum. This, in turn, might facilitate bacterial colonization, cholangitis, or even LA [7]. Consequently, ES might be associated with the development of PLA as described by several authors [4].

Other possible complications of ERCP include bleeding, with a reported incidence rate of approximately 1-2%, which is mostly observed after ES [8]. One-half of UGIB occurs immediately after the procedure, with a delay ranging from 24 hours up to several days observed in some patients. Post-ES bleeding is often mild to moderate in severity, self-limited, and rarely life-threatening [9], except in patients with a bleeding diathesis. Most clinically significant bleeding can be managed with medical and endoscopic therapy, with injection therapy with epinephrine being the most common first-line treatment [10]. In contrast, our patient, presented with post-ES bleeding besides PLA and iatrogenic hemoperitoneum 17 days after ERCP, a very rare timing, with unusual hemodynamic instability, and was refractory to standard endoscopic therapy. Although the hemoperitoneum (stabilized after red blood cell transfusions) may have acted as a confounding factor in terms of the beginning of UGIB and severity of hemodynamic instability, the endoscopic finding of active bleeding along with resolution with the placement of FCSEMS supports our hypothesis. The infection and bacteremia predisposed the patient to sepsis, which was associated with coagulation abnormalities that, even if subclinical, may have had a role in this delayed bleeding. There are rare cases of post-ES bleeding refractory to conventional hemostatic technique [11], and FCSEMS is an effective second-line modality, before resorting to embolization or surgery [10].

## Conclusions

This report is intended to alert physicians to the importance of timely recognition of some complications of commonly performed procedures and provides further evidence of the relationship of ES, PLA, and UGIB with hemodynamic instability. As most of these patients present to the emergency room, every patient with LA should be questioned regarding former procedures that could be linked to PLA. Furthermore, clinicians should maintain a high index of suspicion for major delayed post-ES bleeding in a patient with UGIB and history of ERCP with ES.
